# News and Coming Events

**Published:** 1907-03-09

**Authors:** 


### NEWS AND COMING EVENTS.

The Communal Council of Brussels has decided to build a large hospital at Jette St. Pierre.

The annual meeting of the new Hospital for Women, 144 Euston Road, will be held at the hospital on March 12 at 3 P.M.

The festival dinner of the Midlesex Hospital has been fixed for May 10. H.R.H. the Duke of Connaught has consented to preside.

Guy's Hospital Medical School has received the sum of £1,000 under the will of the late Dr. Oldham, towards the endowment of a special prize in ophthalmology.

Mr. H. Overton Wills, who has given more than £20,000 to Bristol charities, has offered to subscribe £1,000 towards the Bath Hospital Fund, provided a sum of £5,000 is raised for the fund before November next.

The honorary secretaries of King Edward's Hospital Fund for London have received the sum of £10,000 from the executors of the late Mr. John Nicholas, being the amount of a legacy (free of duty) bequeathed by the deceased to the Fund.

The annual meeting of the Governors of the French Hospital will be held at 178 Shaftesbury Avenue, W.C., on March 14, at six o'clock. The French Ambassador, supported by the Lord Mayor and Sheriffs, will preside at the annual banquet on April 20.

During last year a scheme was started at Torbay for collecting from the school children of the town a sum sufficient for the maintenance of a cot in the local hospital to be named the "King Edward Cot." The sum of £35 has been received from the originators of the scheme.

The annual council of Governors of the Great Northern Central Hospital, Holloway Road, N., will be held at the hospital on Thursday, March 14, at 5 P.M. The following ladies have been proposed as members of the Committee of Management: Mrs. Lansdowne Beale, Miss Amy Hill, and Mrs. David Waterlow, in terms of a recommendation from the Committee that three lady Governors be elected to the Committee.

The annual dinner of the Association of Scottish Medical Diplomates was held at the Trocadero Restaurant on Friday last, Dr. St. Aubyn-Farrar presiding. In proposing the toast of "The Association," the Chairman said that the immediate design of the Association might be expressed in three words: "Co-operation, co-ordination, arbitration." There was a good attendance, and an excellent musical programme was rendered.

Just before the commencement of the excavations for the new wing at the Torbay Hospital, Mr. J. Burridge, an old inhabitant of Torquay, informed the contractor for the work that about fifty years ago, whilst tipping rubbish on the site, he lost a silver watch, which he felt sure was somewhere under the earth. On carefully searching the excavated earth the watch, which had been lost for half a century, came to light, and was identified by Mr. Burridge as the one he carried when quite a lad.

The memorial stone of the new south-east wing of Leicester Infirmary was laid on Wednesday last by the Duke of Rutland.

The treasurers of the Royal National Orthopædic Hospital have received a legacy of £500, duty free, under the will of the late Mr. S. W. Fryett.

Under a special by-law, authorising the admission to the Sanatorium of children suffering from "surgical tuberculosis," the Notts Sanatorium last year admitted one child.

Dr. Chas. B. Hall, who has been appointed to the consulting medical staff of the Dewsbury Infirmary, held the post of honorary physician to that institution for twenty-four years.

The annual general meeting of members of the Corporation of the North London and University College Hospital will be held in the Board-room of the hospital on Friday, March 22, at four o'clock.

The banquet in aid of the Railwaymen's Convalescent Home, Herne Bay, will be held in the Hamilton Hall of the Great Eastern Hotel, on Wednesday, March 20, Lord Claude Hamilton, Chairman of the Great Eastern Railway, in the chair.

Lady St. Helier is lending her house, 79 Harley Street, W., for a sale of work in aid of the Ragged School Union and Shaftesbury Society, to be held on Tuesday and Wednesday, March 5 and 6, and to be opened the first day, March 5, at 2.30, by H.R.H. Princess Louise Augusta of Schleswig-Holstein.

Their Royal Highnesses the Prince and Princess of Wales have graciously consented to open the new wards and extensions of the Tottenham Hospital on Tuesday, May 7. H.R.H. the Princess Louise (Duchess of Argyll), the President of the hospital, will be present to receive their Royal Highnesses on this occasion.

The recent resignation by Mr. Willoughby Furner of the post of senior surgeon to the Brighton County Hospital severs a family tie which has existed for an exceptionally long period. Mr. Willoughby Furner has seen great developments in the scope of the hospital's beneficent work during his 30 years' record. He has been appointed consulting physician to the hospital.

Bovril, Limited, are issuing another of their well-known gravures in connection with their Bonus Picture Scheme. This time they have selected Arthur Elsley's "A Tempting Bait," exhibited in the 1906 Academy. The reproduction, measuring 28 by 19½, is on fine art plate paper 40 by 30, and reflects great credit on the publishers, Messrs. C. W. Faulkner and Co.

The Leeds Workpeople's Hospital Fund, which was started some twenty years ago, now stands at £150,281, a magnificent sum when it is remembered that most of it has been contributed in pennies and halfpennies. Of this sum, £118,543 has been contributed to the various medical charities, and over £29,000 has been expended on convalescent homes.

